# An International Survey-based Algorithm for the Pharmacologic Treatment of Irritability in Huntington's Disease

**DOI:** 10.1371/currents.RRN1259

**Published:** 2011-08-30

**Authors:** Mark Groves, Erik van Duijn, Karen Anderson, David Craufurd, Mary C. Edmondson, Nathan Goodman, Daniel P. van Kammen, LaVonne Goodman

**Affiliations:** ^*^Departments of Neurology and Psychiatry, Beth Israel Medical Center, New York, NY; ^†^Department of Psychiatry, Leiden University Medical Centre, Leiden; and Centre for Mental Health Care Delfland, Delft, Netherlands; ^‡^Department of Psychiatry and Department of Neurology, University of Maryland, School of Medicine, Baltimore, MD USA; ^§^University of Manchester, Manchester Academic Health Sciences Centre and Central Manchester University Hospitals NHS Foundation Trust, Manchester, UK; ^¶^Department of Psychiatry, Duke University Medical Center & North Carolina Center for the Care of Huntington's Disease; ^#^Institute for Systems Biology; ^**^CHDI Foundation, Inc., Princeton, NJ and ^††^Huntington's Disease Drug Works

## Abstract

It is generally believed that treatments are available to manage irritability in Huntington’s disease (HD). However, lack of an evidence base prevents the establishment of treatment guidelines for this symptom. The research literature fails to address behavioral intervention strategies, drug selection, drug dosing, management of inadequate response to a single drug, or preferred drugs when additional behavioral symptoms comorbid to irritability are present. In an effort to inform clinical decision-making we surveyed an international group of experts to address these points. The experts consistently endorsed an antipsychotic drug (APD) as first choice for treatment of urgent and aggressive irritability behaviors. However, there was variation in practice patterns for treating less severe symptoms. Serotonin reuptake inhibitors (SSRIs) were first choice drug treatments by most respondents across all geographic regions. However, APDs were also endorsed as first choice for mild or moderate irritability, more frequently in Europe than in North America and Australia. Antiepileptic mood stabilizers (AEDs) were used by fewer respondents as first choice drug. Perceived efficacy for control of mild or moderate irritability was judged somewhat higher for APDs than SSRIs or AEDs. Benzodiazepines were not used as monotherapy, but frequently as an adjunctive drug in the setting of comorbid anxiety. Though many cited lack of experience with mirtazapine, others familiar with its use in HD chose it as an alternative monotherapy, or as adjunctive therapy if insomnia was a comorbid factor. This report presents survey results, reviews available irritability studies, and lastly proposes an algorithm for the treatment of irritability in HD derived from expert preferences obtained through this survey.

## **Introduction**

Huntington’s disease (HD) is a neurodegenerative disorder with an autosomal dominant hereditary pattern, caused by an elongated CAG repeat on chromosome 4 [1]. HD is clinically characterized by motor dysfunction, neuropsychiatric symptoms, and cognitive impairment leading to dementia at end-stage. Onset of clinical symptoms most commonly occurs between the ages of 30 and 50 years, and follows a progressive course with an average disease duration of 20 years. No treatment is available to delay onset or to slow progression of the disease.

 Irritability is a frequently reported neuropsychiatric disorder in HD, in addition to depression, anxiety, apathy, and obsessive-compulsive behaviors [2]. HD irritability has been defined as a temporary psychologic state characterized by impatience, intolerance, and reduced control over temper, which can progress to angry and aggressive verbal or behavioral outbursts [3]. Estimates of irritability vary from 31% to 65% according to studies using different methods of sampling and measurement [4] [5]. A study comparing self and care-partner assessment shows a higher reported incidence for irritability when information is obtained from care-partners [6], suggesting a component of lack of awareness in patients. However, many are aware and recognize that they are too easily provoked, and take more time to calm down after provocation [7]. Irritability can precede the onset of motor symptoms of HD [8] and it occurs over all stages of disease. However, measures of irritability do not track with disease progression [7] [9]. Further, patients differ in the degree to which they experience irritability: some "feel" irritable, some exhibit mild irritability without further behavioral manifestations, while the behavior of others can escalate to physical aggression.

 At all levels of severity, it is vital to recognize irritability as part of the disease process, and not to mischaracterize patients as difficult, stubborn, or “character disordered” which adds to strain experienced by patients and families. Early recognition and treatment of this symptom is important because irritability behaviors that escalate can be a tipping point in terms of care partner burnout and subsequent need to institutionalize HD patients.

**Box 1. Abbreviations for drugs and drug classes**

**Table:** 

AED	mood stabilizing anti-epileptic drug
APD	antipsychotic
BZD	benzodiazepine
CMI	clomipramine
SNRI	serotonin-norepinephrine reuptake inhibitor
SSRI	selective serotonin reuptake inhibitor
TCA	tricyclic

 Though behavioral interventions have not been studied, or drugs officially approved to manage irritability in HD, it is generally accepted that behavioral interventions, APDs, SSRIs, AED's, and other drugs are useful in treatment of these symptoms [10]. However, a recent Cochrane review of symptomatic treatments for HD concluded that no treatment recommendations could be made based on evidence from the research literature [11]. Lacking an adequate evidence base to guide treatment, we surveyed current clinical practice among an international group of HD experts to ascertain practice-based preferences. Recognizing the limits of expert opinion, and with the expectation that future clinical research will provide evidence-based information, we present survey results to provide direction for the management of irritability in HD. 

## **Methods**

The irritability survey was one of three symptom surveys developed by three core groups of nine psychiatrists and neurologists drawn from the European Huntington’s Disease Network (EHDN) and North American Huntington Study Group (HSG), and an HD family representative. Concurrent surveys were developed for obsessive-compulsive behaviors and chorea in HD. These three specific symptoms were chosen by core group consensus as those in greatest need of expert guidance relative to other symptoms of HD, including depression, anxiety, sleep disorder, and psychotic behaviors, for which clinical practice follows guidelines developed for these conditions in the general population. Data on coincident surveys for the treatment of obsessive-compulsive behaviors and chorea are presented in separate reports [12] [13].

 The survey for HD irritability was developed by four core group psychiatrists from different geographic areas who have had extensive experience treating behavioral symptoms in HD. Questions were constructed electronically. Subsequently the survey was sent by email link to a larger international group of 66 EHDN and HSG physician leaders from HD specialty centers in 11 European countries, 10 U.S.A states, 4 Canadian provinces, and 3 Australian states. Experts were selected by the combined EHDN and HSG core groups as being knowledgeable in treating HD symptoms. Follow-up email or telephone reminders were used to encourage survey participation. Respondents were sent a small honorarium after completing the survey.

 The survey consisted of 51 multiple-choice questions with 680 alternative answers, and the option to add comments. Questions addressed respondents' demographics and their patterns of pharmacological treatment. By core group consensus, the survey focused on 5 drug classes (SSRIs, APDs, AEDs, BZDs, and tricyclic antidepressants (TCAs)) and 4 specific drugs (propranolol, clomipramine (CMI), buspirone, and mirtazapine), that have been used to treat HD irritability. In iterative fashion, each drug/drug class was addressed separately through additional questions covering the following: patterns of use (first choice, alternative monotherapy, adjunctive therapy, not an appropriate use, insufficient experience), perceived effectiveness (very effective, effective, somewhat effective, minimally effective), and preferred drugs within each class. Branching logic utilized in the survey prevented the answering of questions if a respondent did not choose a specific treatment as first or alternative monotherapy, or indicated having no experience with a particular treatment.  However, branching logic allowed a respondent to check more than 1 drug or drug class as first or alternative therapy, or to check no drug as first choice.  Questions also covered augmentation and switch strategies, timing of dose titration, and preferred drugs when other behavioral symptoms comorbid to irritability were present.

 Following analysis of survey data, the core group presented results and a proposed algorithm for the treatment of irritability to a broader group of international HD experts attending the 2010 EHDN and HSG meetings for the purpose of obtaining further review. 

## **Results**

 Of the 66 expert clinicians contacted, 55 responded. Not all respondents answered all individual questions. Of these 55 expert respondents, 26 were from Europe, 23 from the United States, 4 from Canada,and 2 from Australia. Most respondents were neurologists (39) or psychiatrists (10). There were 2 respondents who were double boarded in neurology and psychiatry and 2 respondents in neurogenetics. Clinical experience was quite substantial among the surveyed experts: over half reported treating more than 100 HD patients annually. 

**Practice patterns by drug or drug class: **The first set of treatment questions concerned drug selection and was phrased as follows: “Assuming there are no comorbid symptoms to influence your decision, what is your practice pattern with the use of [drug or drug class] for the treatment of irritability in Huntington’s disease?” Drugs/drug classes included SSRIs, APDs, AEDs, BZDs, and tricyclic antidepressants (TCAs)) and 4 specific drugs (propranolol, clomipramine (CMI), buspirone, and mirtazapine. Alternative usage included: first choice, alternative monotherapy, adjunctive therapy, not an appropriate use, or insufficient experience. Interpretation of this first set of questions was complicated by limitations in the survey branching logic which allowed for the respondent to check more than 1 first choice or no first choice. Five respondents checked no first choice, while 2 had more than 1 first choice.  See figures 1-3 and table 1.

**Figure:**
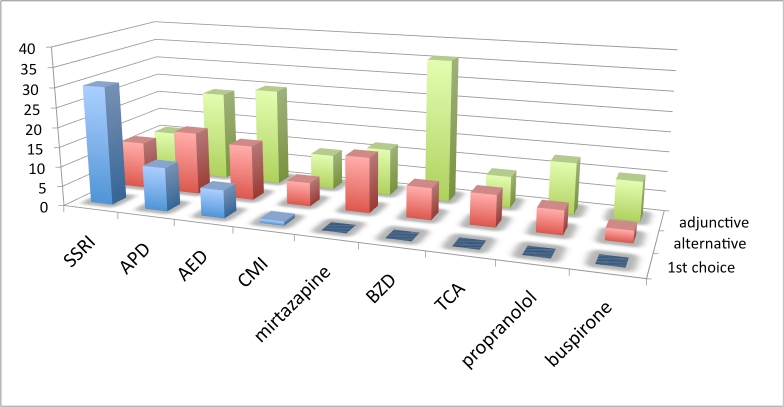
**Figure 1. Choice of irritability drug across all geographic regions. Vertical axis is number of responses. See box 1 for abbreviations.**

**Figure:**
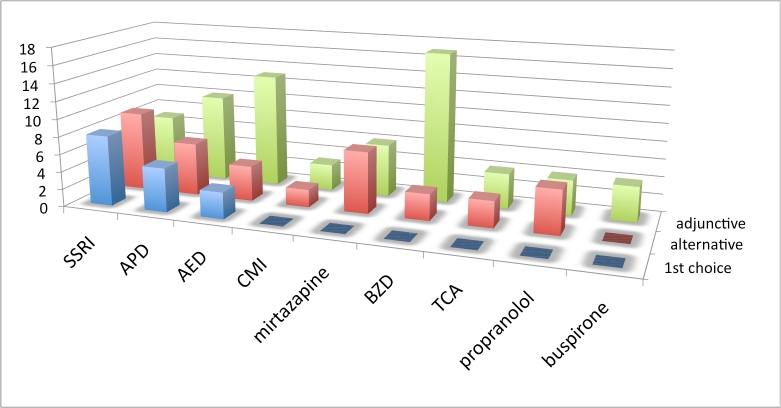
**Figure 2. Choice of irritability drug, European respondents. Vertical axis is number of responses.  See box 1 for abbreviations.**

**Figure:**
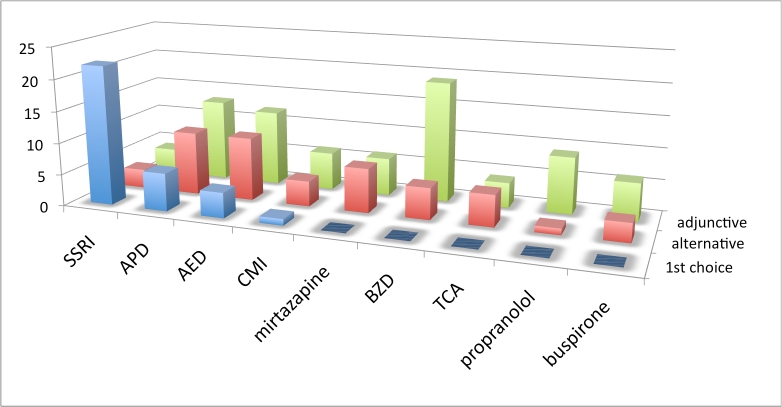
**Figure 3. Choice of irritability drug, North American and Australian respondents. Vertical axis is number of responses.  See box 1 for abbreviations.**

**Table 1. Choice of drug for treating irritability across all geographic regions. N is number of responses. Percentages are relative to N. See box 1 for abbreviations.**

**Table:** 

						
**SSRI**	53	57%	23%	21%	0%	0%
**APD**	52	21%	31%	44%	4%	0%
**AED**	51	14%	27%	49%	0%	10%
**CMI**	51	2%	12%	18%	8%	61%
**mirtazapine**	50	0%	28%	24%	6%	42%
**BZD**	50	0%	16%	72%	10%	2%
**TCA**	50	0%	16%	16%	32%	36%
**propranolol**	50	0%	12%	26%	16%	46%
**buspirone**	50	0%	6%	20%	14%	60%

 Results from this set of questions, when no comorbid symptoms influenced treatment decisions revealed some variation in practice patterns. Across all geographic regions SSRIs were most frequently chosen as the first drug for treating mild to moderate HD irritability. Though a less frequent choice than the SSRIs overall, APD usage for treatment of mild or moderate irritability was more common in Europe than in North America and Australia. AEDs were less frequently endorsed as first choice drug. When combining monotherapy choices (first and alternative), SSRIs were most frequently endorsed (80%), followed by APDs (53%) and AEDs (43%). No respondent chose buspirone, mirtazapine, propranolol or TCAs as first choice drugs, but each was cited as an alternative monotherapy choice by a minority of respondents. Many cited lack of experience with these drugs for treating irritability. The BZDs were most frequently used as an adjunctive drug. Though many (43%) were not familiar with the use of mirtazapine for irritabilty, half of those familiar with the drug chose it as an alternative monotherapy. 

**Perceived efficacy of drug choice: **Most experts indicated that efficacy of APDs was somewhat higher than SSRIs. Table 2 summarizes expert views about the relative efficacy of surveyed drugs. 

**Table 2. Expert opinion of drug efficacy for treating irritability. N is number of responses. Percentages are relative to N. See box 1 for abbreviations.**

**Table:** 

					
**SSRI**	51	14%	43%	37%	6%
**APD**	50	18%	50%	32%	0%
**AED**	45	7%	31%	58%	4%
**CMI**	16	12%	19%	56%	12%
**mirtazapine**	26	0%	19%	77%	4%
**BZD**	45	4%	29%	58%	9%
**TCA**	16	0%	19%	56%	25%
**propranolol**	18	0%	11%	50%	39%
**buspirone**	13	0%	8%	54%	38%

**Perceived benefit of high dose SSRI optimization: **Respondents were also asked about SSRI dosing optimization for treating irritability in HD to upper limits of manufacturer recommended dosage for depression. Though all respondents perceived a level of increased effectiveness with higher dosing, the degree of effectiveness varied widely. Subsequent to the survey, the Federal Drug Administration issued a directive to change manufacturer recommended high dosage of citalopram from 60 mg to 40 mg per day, based on an increase in heart arrhythmias, and lack of larger dose benefit in treating depression.  However, for treatment of irritability, six respondents reported beneficial result in selected patients exceeding the highest dose recommended for depression.****

**Dosing interval choices: **Respondents were asked about dosing titration intervals for the drug/drug class alternatives. Preferred titration intervals varied greatly within each drug or drug class. See table 3. 

**Table 3. Choice of dosing titration intervals for drugs used to treat irritability. N is number of responses. Percentages are relative to N. See box 1 for abbreviations.**

**Table:** 

					
**SSRI**	52	12%	33%	44%	12%
**APD**	49	33%	33%	24%	10%
**AED**	45	18%	42%	33%	7%
**CMI**	16	6%	12%	75%	6%
**mirtazapine**	26	8%	50%	35%	8%
**BZD**	44	55%	20%	11%	14%
**TCA**	17	0%	41%	47%	12%
**buspirone**	13	15%	31%	46%	8%

**Adding or switching drugs for inadequate response to initial drug choice: **The next set of iterative questions regarded strategies for adding or switching drug when an initial drug choice failed to adequately treat irritability in HD in the setting of no comorbid symptom to influence choice. The most notable result to this set of questions is that no consistent pattern was demonstrated for either SSRI or APD, the preferred initial choices. When SSRI was chosen as initial monotherapy but gave no or only partial benefit, the experts reported adding: an APD (30%), AED (16%) or BZD (7%), or switching to another SSRI (14%), or switching to an APD (12%), AED (7%), or BZD (5%). Similarly when an APD was chosen as initial monotherapy and did not give adequate response, the experts reported switching to another APD (28%), adding an SSRI (15%), AED (15%) or BZD (11%), or switching to an AED (8%) or SSRI (6%).  

**Table 4. Alternate choice of drug for treating irritability when inadequate response to initial therapy. N is number of responses. Percentages are relative to N. Alternate listed only if chosen by 5% or more of respondents. See box 1 for abbreviations.**

**Table:** 

			
**SSRI**	43	add APD	30%
		add AED	16%
		switch to another SSRI	14%
		switch to APD	12%
		switch to AED	7%
		add BZD	7%
		switch to BZD	5%
**APD**	36	switch to another APD	28%
		add SSRI	19%
		add AED	19%
		add BZD	11%
		switch to AED	8%
		switch to SSRI	6%

**Specific drugs favored within class: **Preferred drugs within class for use in HD irritability included the SSRIs citalopram (35%), sertraline (25%) and paroxetine (15%); the APDs olanzapine (51%), risperidone (32%), and quetiapine (30%). Sulpiride and tiapride available only in parts of Europe were preferred by 7 European respondents. Insufficient experience was reported by many for clozapine, aripiprazole, ziprasidone, and pimozide. A high percentage of respondents (83.7%) preferred second-generation over first-generation antipsychotics. AED preferences included valproate derivatives (74%) and carbamazipine (23%), with a few others choosing each of the following: lamotrigine, gabapentin, topiramate and levetiracetam. 

**Other choices: **The mood stabilizer lithium was an option for treating HD irritability. Only one expert rated lithium to be his/her first choice, but a substantial number of experts (23.6%) had no experience in using lithium for HD irritability, and 17.4% rated it as inappropriate. Only one respondent reported using benzodiazepines (BDZs) as first choice of treatment for irritability in HD. Most respondents prescribed BDZs as adjunctive therapy (70.6%). No one endorsed propranolol as a first-choice medication, 6 chose it as alternative monotherapy, and 13 as adjunctive therapy for irritability in HD. Almost half of the experts reported insufficient experience with propranolol for this indication.

**Preferred medication choices for irritability with comorbid psychiatric symptoms: **Given comorbid depression, anxiety, or perseverative behaviors, an SSRI was the first choice of the experts for treating HD irritability. Antipsychotic drugs were the first choice when comorbid psychosis, aggression, impulsivity, or hypersexuality were present. When patients who were irritable also suffered from insomnia, the favored medication choices were BDZs or mirtazapine.  See figure 4 and table 5.  

**Figure:**
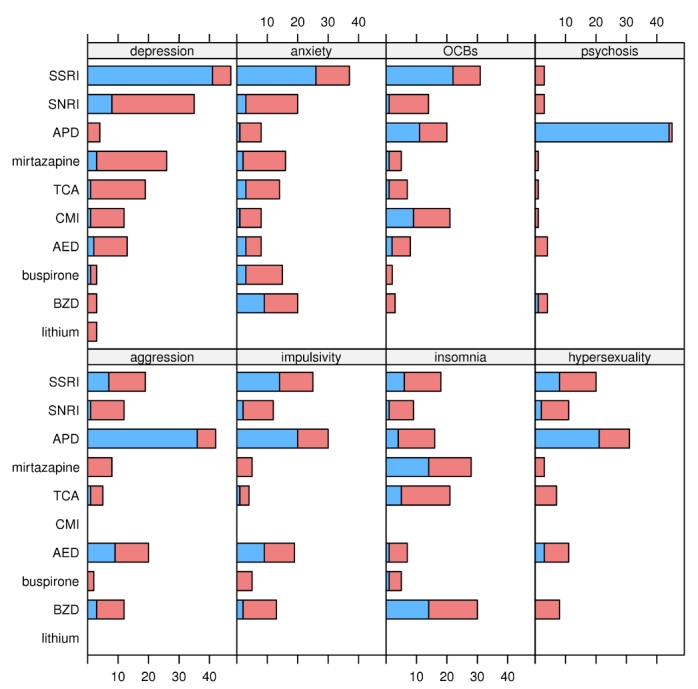
**Figure 4. Choice of drug for** **irritability that occurs with a given comorbid symptom. Blue bars indicate the number of experts who selected the drug as first choice; red bars indicate the number who selected the drug as alternative monotherapy. See box 1 for abbreviations.** 

**Table 5. Choice of drug for treating irritability that occurs with a given comorbid symptom. Percentages are relative to the number of experts who provided information for any symptom x drug combination (47). The last column is the sum of the previous two; the percentages do not always match precisely because of roundoff. The table only includes drugs chosen by 10% or more of the experts. See box 1 for abbreviations.**

**Table:** 

				
**depression**	**SSRI**	87%	13%	100%
	**SNRI**	17%	57%	74%
	**mirtazapine**	6%	49%	55%
	**TCA**	2%	38%	40%
	**AED**	4%	23%	28%
	**CMI**	2%	23%	26%
**anxiety**	**SSRI**	55%	23%	79%
	**BZD**	19%	23%	43%
	**SNRI**	6%	36%	43%
	**mirtazapine**	4%	30%	34%
	**buspirone**	6%	26%	32%
	**TCA**	6%	23%	30%
	**AED**	6%	11%	17%
	**APD**	2%	15%	17%
	**CMI**	2%	15%	17%
**OCBs**	**SSRI**	47%	19%	66%
	**CMI**	19%	26%	45%
	**APD**	23%	19%	43%
	**SNRI**	2%	28%	30%
	**AED**	4%	13%	17%
	**TCA**	2%	13%	15%
	**mirtazapine**	2%	9%	11%
**psychosis**	**APD**	94%	2%	96%
**aggression**	**APD**	77%	13%	89%
	**AED**	19%	23%	43%
	**SSRI**	15%	26%	40%
	**BZD**	6%	19%	26%
	**SNRI**	2%	23%	26%
	**mirtazapine**	0%	17%	17%
	**TCA**	2%	9%	11%
**impulsivity**	**APD**	43%	21%	64%
	**SSRI**	30%	23%	53%
	**AED**	19%	21%	40%
	**BZD**	4%	23%	28%
	**SNRI**	4%	21%	26%
	**mirtazapine**	0%	11%	11%
	**buspirone**	0%	11%	11%
**insomnia**	**BZD**	30%	34%	64%
	**mirtazapine**	30%	30%	60%
	**TCA**	11%	34%	45%
	**SSRI**	13%	26%	38%
	**APD**	9%	26%	34%
	**SNRI**	2%	17%	19%
	**AED**	2%	13%	15%
	**buspirone**	2%	9%	11%
**hypersexuality**	**APD**	45%	21%	66%
	**SSRI**	17%	26%	43%
	**AED**	6%	17%	23%
	**SNRI**	4%	19%	23%
	**BZD**	0%	17%	17%
	**TCA**	0%	15%	15%

## **Discussion**

 Though irritability is common in HD and several medication classes are employed in clinical practice to treat irritability in HD, only a few small studies are available. Further, these studies are hard to compare, with varying definitions of irritability and measurement tools utilized, and most patients in these reports were using additional medications which may have influenced results. Among these studies are: a single report on the use of an SSRI [14], a few other studies on various APDs, olanzapine alone [15], olanzapine and valproate [16], another on haloperidol and lithium [17], and more recently on nabilone, a cannabinoid most often used in treatment of nausea caused by cancer chemotherapy [18]. All of these studies represent a low level of evidence and give little guidance for treatment.

**Box 2. Key behavioral interventions for managing irritability.**

**Table:** 

**Expectations**	Care partners should have appropriate expectations regarding a patient's abilities and needs. Some HD patients with high levels of symptoms have great difficulty controlling irritability behaviors and should not be expected to consistently control symptoms.
**Prevention**	If there are situations that trigger irritability behaviors (e.g., discussing driving ability or cigarette smoking), it is best to avoid these topics. It is best to set and maintain regular schedules and routines. Identify and address sources of physical and emotional distress such as pain, hunger, thirst, difficulty with communication, frustration with failing abilities, boredom and unexpected change in routine.
**Redirection**	Redirection is the most common environmental strategy used. It may take the form of changing the subject, starting a new activity, moving to a different room, placing an interesting object (e.g., a coin) in the patient's hand as a distraction, and the like.
**Calm Response**	Confronting or arguing with the patient may escalate irritability severity to aggressive levels. Any discussion of the event triggering this behavior should wait till the patient is calmed.
*Source*: LEARNet Tutorial on Anger And Anger Management	

In an earlier survey (results not reported) experts agreed that behavioral intervention that includes education for patients and their families on practical behavioral approaches is recommended prior to, and to accompany pharmacological interventions. Though there is no formal guidance on how to use these interventions in HD, we recommend several strategies, based on treatments used in traumatic brain injury.  Box 2 summarizes helpful recommendations. It is important to address triggers and recommended behavioral strategies at each patient and care-partner visit.

 Overall, the present survey reveals a variety of pharmacological treatment patterns, without clear consensus in many areas. Nevertheless, some practice patterns emerge as preferred by many of the experts, with SSRIs and APDs as the most favored medication classes.

 When the irritability is severe and/or there is an urgent need for treatment, the experts favored starting either an atypical APD, or less frequently an AED such as valproate. When irritability in HD is not accompanied by psychotic symptoms and is not severe or urgent, an SSRI was favored as first choice of treatment. No consensus existed among the experts as to the duration of treatment required before onset of full clinical effect, but clinical experience would suggest the onset of SSRI benefit for irritability in HD occurs earlier than benefit for depression, so a range of 2 to 4 weeks was chosen by the authors to emphasize this point in the algorithm. Assessment of adherence is always important when there is an inadequate response.  This is emphasized in the algorithm, as is dose optimization when lower doses produce only a partial response in treating irritability.

 The experts widely agreed that an SSRI is the first choice for treating HD irritability when accompanied by depression. When residual depressive symptoms remain after optimizing the SSRI in an irritable HD patient, we recommend optimizing treatment of the depression first, before proceeding to augmentation or switch strategies for managing irritability. When irritability in HD is accompanied by impulsivity, aggression, or hypersexuality, most experts favored use of atypical APDs or less frequently AEDs. This finding is indicated in the decision node that directs clinicians to favor these two classes among the four medication classes recommended for combination therapy when monotherapy with an SSRI is insufficient to achieve a satisfactory response to irritability.

 The use of second-generation APDs and AED drugs were favored over propranolol and BZDs, due to their greater perceived effectiveness. Few survey respondents use propranolol, despite its wide use in other neuropsychiatric settings such as traumatic brain injury [19]. A Cochrane review of pharmacologic treatment of agitation in acquired brain injury concluded that Beta-blockers had best efficacy [20].  Only a few survey respondents use, or were familiar with, buspirone for HD irritability despite early positive research reports [21] [22].  If two or more consecutive combination trials across at least two distinct classes fail to achieve a full response to irritability (with adequate dose, duration and adherence), referral to a specialist is recommended.

 Based on the results of this international expert survey, a clinical practice algorithm for the treatment of irritability in HD was constructed. The authors do not mean to imply that following the steps most often chosen by experts will result in best outcomes. Treatment response varies greatly in HD, and is particularly hard to predict. The steps represented in the algorithm are meant to guide, not decide any individual's treatment.  Only the clinician can address the complexities of any specific patient, where treatment must be tailored to fit individual needs.  

## **Algorithm**

 (Click on the figure below for a printable, single page view of the algorithm). 

**Figure:**
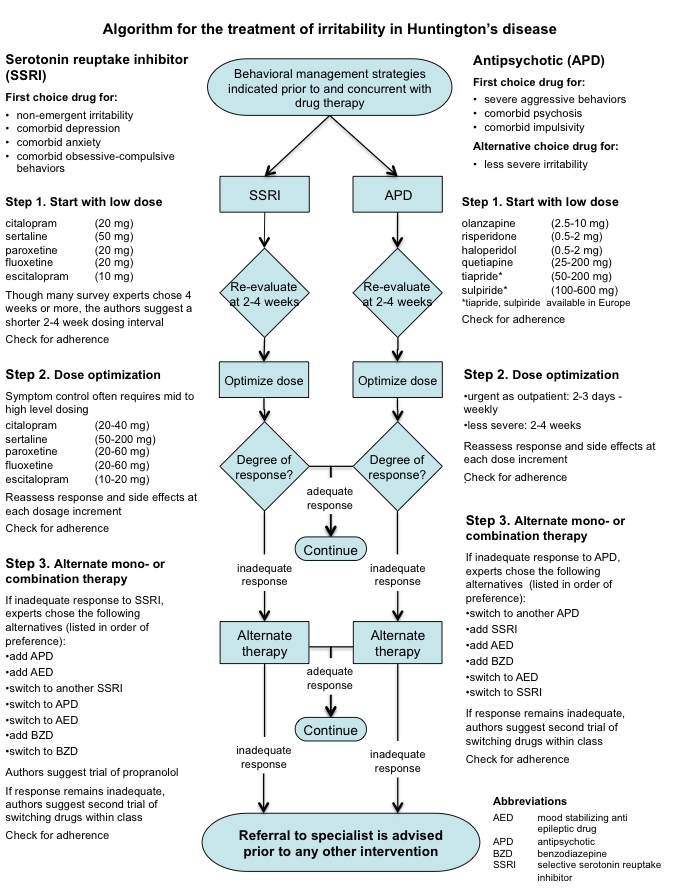

## **Conclusions**

 Based on survey outcomes the experts agreed that behavioral interventions and education for patients and care partners are helpful in management of irritability in HD. There was good agreement among the experts that an an APD is first choice when irritability is expressed as aggressive behaviors. For less severe irritability, most of experts in the combined regions chose an SSRI as first choice drug.  APDs were more often chosen as first choice drugs in Europe than North America and Australia for less severe irritability  The SSRIs and APDs were perceived as similar in efficacy for treating non-urgent irritability, and each of these options was used to augment the other. AEDs were not frequently utilized as monotherapy, but were used as augmenting agents to both SSRI or APD when each was ineffective alone. There was agreement that BZDs are not substantially effective as monotherapy, but agreement on their use as adjunctive therapy, particularly when anxiety is a comorbid factor. 

 The results of the survey point out the need for further study of irritability in HD. Development of a standard definition of these symptoms in HD, along with a validated scale for assessment, would greatly advance understanding of these conditions. Review of the literature shows there is a pressing need for treatment studies to determine which psychopharmacological and behavioral treatments are most efficacious. Head to head comparisons of the most frequently used agents and augmenting strategies would provide practical information. 

**Limitations:** Survey results are not a substitute for evidence-based study. Instead, these results present treatment options based on a synthesis of opinions from a large group of experts. However, selection of the experts surveyed was not systematic. The core group authors generated a list of expert clinicians based on their personal knowledge of individuals active in the clinical research networks.   A systematic survey of all members of the EHDN and HSG, though less feasible, would have provided a larger and more diverse sample. Further, as shown in this survey, practice patterns were influenced by geographic location.

 A potential limitation is the inherent design of this survey that asked experts to consider irritability as an isolated symptom, which is not a common presentation in clinical practice. Recall bias may also have occurred, with survey results limited by the accuracy of respondents’ recall, with potential for over- or underestimation of drug efficacy. SSRIs were listed first in the questionnaire, reflecting the authors’ own views but possibly biasing answers toward this class of medication. A random order of presentation for medication class, varied among participants, would have been methodologically more sound. Though we believe the survey questions were comprehensive, they did not cover every possibility and may have omitted other useful queries.

 This project received funding support in part by Lundbeck Inc., an arrangement that could introduce bias. In an effort to limit this bias, HSG and EHDN core committee members and survey respondents had no knowledge of Lundbeck Inc. support during the survey process or data analysis.

**Acknowledgements**

The authors thank those HD experts who shared knowledge and participated in this survey: Karen Anderson, Tomasin Andrews, Anna Rita Bentivoglio, Kevin Biglan, Jodi Cori-Bloom, Raphael Bonelli, Jean-Marc Burgunder, Jang Ho Cha, Edmond Chiu, Peter Como, David Craufurd, Merit Cudkowicz. Matthias Dose, Richard Dubinsky, Erik van Duijn, Alexandra Durr, Mary Edmondson, Andy Feigen, Joaquim Ferreira, Mark Groves, Arvid Heiberg, Don Higgins, Stephen Hersch, Joseph Jankovic, Hans Jung, Karl Kieburtz, Barry Kremer, Pierre Krystkowiak, Martin Kucharik, Blair Leavitt, Ann Catherine Bachoid-Levi, Wayne Martin, Elizabeth McCusker, Ann Messer, Marsha Nance, Michael Orth, Oksena Osuchowersky, Susan Perlman, Asa Petersen, Josef Priller, Hugh Rickards, Raymund Roos, Adam Rosenblatt, Diana Rosas, Ann Rosser, Jan Roth, Burton Scott, Kathleen Shannon, Shiela Simpson, Ira Shoulson, Nicholas Stoy, Sarah Tabrizi, Francis Walker, Eric Wexler, Vicki Wheelock, Daniel Zielonka. 

 Further gratitude to Dr. Richard Dubinsky and Dr. Eric Wexler who provided expert advice in survey creation,  CHDI Foundation for expert advice and technical assistance, and Ann Covalt for editorial assistance.

**Funding sources  **The Huntington’s Disease Society of America (HDSA), Huntington Society of Canada (HSC), European Huntington’s Disease Network (EHDN), and HD Drug Works (HDDW) provided funding for this project. Support from Lundbeck Inc. was provided by a one time unrestricted grant to HDDW. The combined funds from HDSA, HSC, EHDN, and HDDW provided stipend reimbursement for expert participation. To prevent bias, experts were kept unaware of the Lundbeck Inc. grant during survey and analysis portions of this project.  

**Competing interests**  Dr. Goodman received unrestricted grant and consultant fees from Lundbeck, Inc. In 2009.  Dr. Edmondson has received consultant fees from Lundbeck, Inc.

**Author roles**

- Drs. Mark Groves (HSG) and Erik van Duijn (EHDN) shared equally in leading the construction and review of the survey questionaire, review of data analysis, writing of first draft and review of manuscript.
- Drs. Karen Anderson and David Craufurd. Construction and review of survey questionnaire.  Review of data analysis and manuscript.
- Dr. Mary Edmondson.  Review of data analysis and manuscript.
- Dr. Dan van Kammen.  Expert adviser. Review of data analysis and manuscript.
- Dr. Nathan Goodman.  Data analysis.
- Dr. LaVonne Goodman. Conception, organization and facilitator for execution of the project. Review of manuscript.
